# Sex-Specific Cut-Offs for High-Sensitivity Cardiac Troponin: Is Less More?

**DOI:** 10.1155/2019/9546931

**Published:** 2019-02-05

**Authors:** Giulio Francesco Romiti, Roberto Cangemi, Filippo Toriello, Eleonora Ruscio, Susanna Sciomer, Federica Moscucci, Marianna Vincenti, Clara Crescioli, Marco Proietti, Stefania Basili, Valeria Raparelli

**Affiliations:** ^1^Department of Internal Medicine and Medical Specialties, Sapienza–University of Rome, Rome, Italy; ^2^Division of Cardiology, San Paolo Hospital, Department of Health Sciences, University of Milan, Milan, Italy; ^3^Department of Cardiovascular and Thoracic Sciences, Catholic University of the Sacred Heart, Rome, Italy; ^4^Department of Cardiovascular, Respiratory, Nephrology, Anesthesiology and Geriatric Sciences, Sapienza–University of Rome, Rome, Italy; ^5^Department of Movement, Human and Health Sciences, University of Rome “Foro Italico”, Rome, Italy; ^6^Istituto di Ricerche Farmacologiche Mario Negri IRCCS, Milan, Italy; ^7^Department of Experimental Medicine, Sapienza–University of Rome, Rome, Italy; ^8^Center for Outcomes Research and Evaluation, Research Institute, McGill University Health Centre, Montreal, Quebec, Canada

## Abstract

Management of patients presenting to the Emergency Department with chest pain is continuously evolving. In the setting of acute coronary syndrome, the availability of high-sensitivity cardiac troponin assays (hs-cTn) has allowed for the development of algorithms aimed at rapidly assessing the risk of an ongoing myocardial infarction. However, concerns were raised about the massive application of such a simplified approach to heterogeneous real-world populations. As a result, there is a potential risk of underdiagnosis in several clusters of patients, including women, for whom a lower threshold for hs-cTn was suggested to be more appropriate. Implementation in clinical practice of sex-tailored cut-off values for hs-cTn represents a hot topic due to the need to reduce inequality and improve diagnostic performance in females. The aim of this review is to summarize current evidence on sex-specific cut-off values of hs-cTn and their application and usefulness in clinical practice. We also offer an extensive overview of thresholds reported in literature and of the mechanisms underlying such differences among sexes, suggesting possible explanations about debated issues.

## 1. Background

Chest pain is one of the most common symptoms and reasons for admission in patients who present to the Emergency Department (ED) [1, 2], setting a major challenge for emergency physicians due to the large number of conditions included in the differential diagnosis [3, 4]. These include cardiovascular diseases (e.g., stable angina, acute coronary syndrome (ACS), aortic dissection, and pulmonary embolism) as well as a broad spectrum of non-cardiovascular causes, such as pneumonia, pleuritis, gastrointestinal disease, and psychogenic causes [5, 6].

In this setting, the first and most important diagnosis to exclude is ACS, due to its high rates of morbidity and mortality [7, 8] and to the need for a prompt therapeutic intervention in case of a confirmed myocardial infarction (MI) [9, 10]. Cardiac troponin (cTn), a protein involved in cardiomyocyte contraction, is a reliable and widely used biomarker of cardiac injury. Its measurement plays an essential role in the diagnostics of ACS [11], to the point of its being included in the universal definition of MI [12]. The availability of a both sensitive and specific marker of myocardial injury, especially with the introduction of the newest high-sensitivity assays (hs-cTn), has revolutionized the workup of ACS in the ED. With this comes the newfound ability to rule out suspected ongoing ischemic heart disease in patients presenting with chest pain and no obvious electrocardiographic signs of MI [13].

To date, a concentration of hs-cTn above the assay-specific upper reference limit (derived from a reference population) is used as a cut-off point for the diagnosis of MI [12]. However, the application of one standard threshold value may not be appropriate for all patients. Sex is one of the several variables that could influence its concentration and interpretation, potentially leading to underdiagnosis and inequality in the treatment of acute MI in women. Coronary artery disease (CAD) and MI are primary causes of mortality in the female population [14]. This is partly due to the frequent atypical clinical presentation in this group, which complicates recognition of symptoms, and can delay following interventions. Moreover, a recent study has shown that women with MI suffer from higher excess mortality compared to men, a difference which is reduced after adjusting for the use of guideline-indicated care [15].

The aim of this review is to summarize the available evidence on the influence of sex on the diagnostic performance of hs-cTn and to present novel implications and applications of sex-specific cut-offs in the management of ACS. For this purpose, we searched for relevant articles on PubMed, combining the terms “troponin”, “hs-cTn”, “gender”, “sex”, “women”, “females”, “men” and “males”.

## 2. Cardiac Troponin: Silver Bullet in the Diagnostics of ACS and MI

The troponin complex is a well-known component of the skeletal and cardiac muscles and plays a key role in myocyte contraction. The complex is composed of three subunits (troponin C, troponin I, and troponin T), each with a peculiar function in the genesis of contraction [16]. Unlike the C subunit, troponins I and T are expressed in the heart in cardiac-specific isoforms (cTnI and cTnT, respectively), allowing them to be recognized as belonging to cardiomyocytes. Following ischemic and non-ischemic myocardial injury, plasmatic levels of both cTnI and cTnT begin to increase and become detectable [11], with kinetics that mostly depend on the type of damage and, in the case of ischemic injury, on the duration of the ischemia and the timing of reperfusion [17]. Usually, troponin levels begin to increase 2 to 4 hours after an ischemic event and remain high for as long as 14 days [18]. Because of these characteristics, cTnI and cTnT have established themselves as the main biomarkers used in the diagnostics of ACS and MI [11, 12]. The advent of hs-cTn has led to an improved ability in detecting slight increases or variations in troponin blood levels, thus resulting in a better chance of rapidly identifying a higher number of MI [19]. Simultaneously, hs-cTn have also increased the safety and reliability of ruling out those patients with stable, low concentrations of hs-cTn and an unlikely ongoing MI [20, 21].

One of the most important open issues regarding the use of hs-cTn is the biological variability in baseline troponin levels, and how this could impact their role in the diagnostics of ACS [19]: 99^th^ percentile levels of hs-cTn are broadly used as the cut-off to rule in or rule out possible MI. These are obtained by studying reference populations composed of supposedly healthy people, but questions were raised about the suitability of using a single cut-off in a heterogeneous real-world population in which patients differ in age, sex, and comorbidities [19, 22]. Some authors argue that serial measurements of hs-cTn could lead to an enhanced prognostic value of this marker by detecting relevant changes in its levels [23–25], thus highlighting the importance of weighting intra-patient variability for the interpretation of hs-cTn values. Indeed, a growing number of studies suggest that the use of a single threshold for hs-cTn irrespective of age, sex, and other parameters may not be ideal [22, 26–28].

Furthermore, several concerns were raised about the definition of hs-cTn, with an ensuing struggle to state unequivocal criteria to identify the necessary standard to be met by an assay in order to be labelled as “high-sensitivity” [29–31]. Consensus of experts proposed a definition that identifies cTn assays as “high-sensitivity” if two criteria are met: (a) total imprecision (i.e., coefficient of variation) ≤10% at the value of the 99^th^ percentile; (b) ability to measure levels of cTn between limit of detection and 99^th^ percentile in at least 50% of healthy subjects [29, 32, 33].

## 3. Sex and Gender: One Key Factor to Consider When Dealing with Troponins 

In the context of cardiovascular disease, several differences between men and women have been described [34]. As for the diagnosis of cardiovascular disease, concentrations of several biomarkers were found to be influenced by sex [35–39], including hs-cTn [40–43], with men reportedly presenting higher concentrations than women. Accordingly, the need for sex-specific reference values has been pointed out by several authors [44–47], while other studies indicate that adopting sex-specific reference intervals for other biomarkers, such as total creatine kinase (CK) activity and MB fraction of CK [48], could also have potential benefits. The same applies to natriuretic peptides [36, 49–53], growth hormone [54], galectin-3 [55, 56], soluble ST2 [57, 58], and proneurotensin [59, 60], supporting the idea that sex differences should be taken into account when approaching laboratory tests.

The first cTn assays, however, required the use of a single, universal cut-off value [61]. The development of hs-cTn assays, in addition to increasing analytical sensitivity, has shown that men present significantly higher concentrations than women for both hs-cTnT and hs-cTnI, highlighting that the upper reference limit for the diagnosis of MI could be two-fold in men compared to women, regardless of the assay being used [29, 44, 61–63].

**Figure 1:**
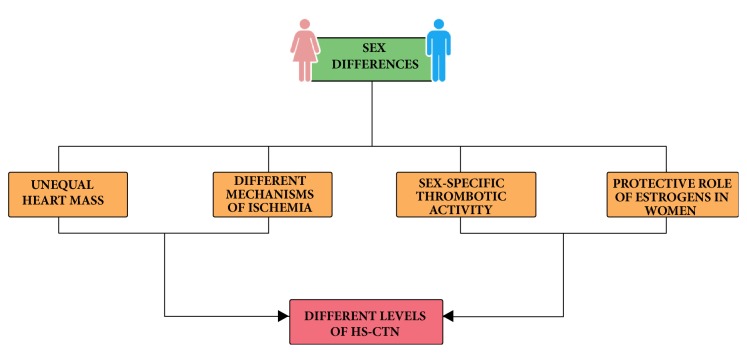
Mechanisms contributing to the discrepancy in hs-cTn levels between men and women.

While still far from being comprehensively understood, several mechanisms may contribute to the aforementioned discrepancy between men and women (Figure 1). Based on the fact that troponin is a measure for the amount of damaged myocardium, some evidence suggests that differences in plasmatic levels of hs-cTn could be attributed to sex-specific variations in body composition [64], cardiac mass [65, 66], and rate of cardiomyocyte apoptosis due to cardiac remodeling [67]. Some insight was provided by authors who outlined potential mechanisms of troponin shedding in the absence of overt membrane injury: variations in the regulation of these events may partially explain the observed variability across healthy subjects [68]. Myocardial response to ischemia and reperfusion is assumed to be unequal in men and women, as well as the pathophysiological mechanism of cardiac ischemia, the grade of coronary atherosclerosis, and the presence of collateral blood flow [69–71]. Sexual hormones may also play a role in the differential expression of hs-cTn levels. Estrogens are thought to exert a protective role on the myocardium: their antioxidant properties and their ability to scavenge reactive oxygen species may contribute to limit cardiomyocyte injury [43, 72–74].

## 4. Sex-Related Cut-Offs: State of the Art

**Figure 2:**
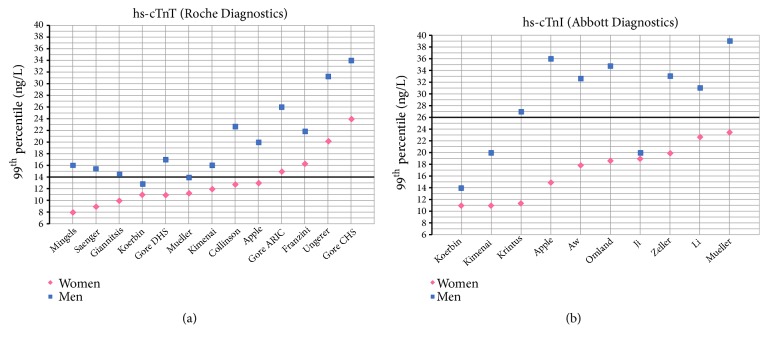
Chart showing different 99^th^ percentile values for hs-cTnT (panel a) and hs-cTnI (panel b) assays, derived from selected population studies as reported in Tables 1 and 2. Bold lines represent non-sex-specific, standard cut-offs for hs-cTnT and hs-cTnI (14 ng/L and 26 ng/L, respectively).

The 99^th^ percentile reference limit (14 ng/L) for hs-cTnT assay (Roche Diagnostics) was set by a study of over 600 apparently healthy volunteers and blood donors [62] and subsequently restated in a multicenter cohort study [75]. In both studies, 50% of the population was composed of females and women showed significantly lower 99^th^ percentile concentrations of hs-cTnT compared to men (10.0 versus 14.5 ng/L and 8.9 versus 15.5 ng/L, respectively). Several other studies support the existence of a discrepancy between 99^th^ percentile values of hs-cTnT in men and women (Table 1 and Figure 2-panel a). Another large study, based on three wide cohorts, reports sex-related critical differences in reference values of hs-cTnT [44], and an Italian-based study of 1600 healthy subjects confirmed the lower threshold for the 99^th^ percentiles in females, with the discrepancy consistent in each age-class [76]. This trend is strongly supported by several other studies, although reference values differ substantially between populations, thus highlighting the impact of the cohort's characteristics [40, 61, 77–82]. Criteria used for the identification of “healthy” individuals are among the most important matters of concern when recruiting a reference population for the purpose of identifying reference values. An elegant study sheds light on how these factors could affect the process of setting a standard reference limit: subsequent application of stricter selection criteria resulted in a progressive reduction of 99^th^ percentile values in a cohort of supposedly healthy people [83], thus addressing the need to implement laboratory tests and clinical assessments in the process of identifying a reference population. These findings are consistent with those observed in other studies [40, 44] and highlight the importance of taking patients' variables into account when dealing with troponins.

Unlike hs-cTnT, several hs-cTnI assays have been developed [84]. The 99^th^ percentile reference values, limits of detection and variance coefficients all vary between assays [19]. Despite these major differences, and consistently with data on hs-cTnT, several studies identified sex-related differences in reference limits of hs-cTnI (Table 2 and Figure 2-panel b). 99^th^ percentile reference values of hs-cTnI were found to be systematically lower in females, regardless of the assay used, ethnicity of the population, or criteria used to identify healthy cohorts. Still, these factors heavily affect the point estimates of the 99^th^ percentile, which differ across the studies [27, 61, 63, 78, 80, 85–90].

## 5. Application of Sex-Specific Cut-Offs in Clinical Practice 

While there is a considerable body of evidence to support the role of sex in influencing reference levels of troponin, no definitive data are available on how this discrepancy could affect the diagnostic and prognostic value of hs-cTn in the work-up of ACS. A synopsis of the studies assessing the prognostic performance of sex-specific cut-offs is reported in Table 3. Specifically, the impacts of three sex-specific cut-offs for hs-cTnT, as reported by Saenger et al. [75], Gore et al. [44], and Kimenai et al. [78], were evaluated in a cohort of patients recruited in an ongoing trial (n=2734, 32% women), each presenting with suspected acute MI. Women were significantly older than men (median age [IQR]: 68 [55-77] versus 59 [48-71]) and showed lower estimated glomerular filtration rate values, whilst higher rates of CAD history and smoking were reported in men. With the application of sex-specific cut-offs instead of the universal one, reclassification from unstable angina (UA) to non-ST elevation MI (NSTEMI) occurred in two women, while only one man was downgraded to UA from NSTEMI. Similar findings were reported with all three sex-based cut-off values analyzed. Reclassification was not shown to impact short-term or long-term prognosis in this cohort, thus not providing evidence in favor of the application of sex-specific thresholds in the diagnostics of ACS [91]. These findings are supported by a subanalysis of the TRAPID-AMI (The High Sensitivity Cardiac Troponin T Assay for Rapid Rule-out of Acute Myocardial Infarction) study, which enrolled over 1200 patients (37% women) with chest pain to assess whether the application of Saenger's sex-oriented cut-offs for hs-cTnT would lead to a better reclassification of MI and an improvement in prognosis. While the use of different cut-offs resulted in an increase of acute MI rates in females (from 16.6% to 22.6%) and a decrease in males, this did not produce any benefit in terms of outcomes [92]. Furthermore, a large retrospective study showed slightly higher rates of diagnostic reclassification (8,4%) and an increase (+3.3%) in MI prevalence in women when using sex-specific cut-offs. Although this study confirmed no advantage in risk prediction when using sex-specific reference values, the risk in women was increased at levels of 10-12 ng/L, which is below the set standard point of 14 ng/L [93]. A recent observational study, focused on the diagnostic performance of several sex-specific hs-cTnT cut-offs for the rule-out of MI, showed an improved specificity with the adoption of different threshold levels [94]. These findings were also consistent with a recent Chinese study in which sex-specific cut-offs were calculated in an original reference population and then further stratified according to age. This study reports an increased specificity for sex-related hs-cTnT thresholds in the diagnostics of AMI, as well as higher negative and positive predictive values [95]. However, the impact of age-stratification probably played a decisive role in this study, still highlighting a possible interplay between these two variables.

The recently published High-STEACS (The High Sensitivity Cardiac Troponin T Assay for Rapid Rule-out of Acute Myocardial Infarction) study reports some of the most interesting findings to date on the topic of sex-specific cut-offs for hs-cTn and on the potential magnitude of the impact which their implementation could have in the management of patients with suspected ACS. In this multicenter, randomized control trial a high sensitivity (hs-cTnI) and a contemporary (cTnI) assay were compared in the diagnosis of suspected ACS. In the first phase of the study, clinical decisions were made according to the cTnI values, while the hs-cTnI concentration was masked. In the second phase, clinicians were provided with the hs-cTnI levels, while cTnI values were masked. The 99^th^ percentiles for hs-cTnI were set to 34 ng/mL and 16 ng/mL in men and women, respectively. Compared with the contemporary assay, reclassification occurred in a significant part (17%) of the myocardial injuries identified by the hs-cTnI, with twofold frequency in women compared to men. However, no significant differences were observed in 1-year outcomes among reclassified patients treated according to cTnI versus hs-cTnI levels [96]. These findings are consistent with a multicenter observational study by Cullen et al., the first large investigation reporting the effects of sex-specific cut-offs (34 ng/L for males and 16 ng/L for females) on prediction of Major Adverse Cardiac Events (MACE) in ED patients. This study suggests that the use of sex-specific reference values for hs-cTnI improves the identification of women at high risk for cardiovascular events within 1 year. Even so, the authors conclude that the net effect across the whole ED population with chest pain symptoms would be minimal and there may be an increased risk of nonidentification of males at high risk for cardiovascular events. The limitation of the study, however, was the use of an overall cut-off to adjudicate endpoints. Overcoming this limitation would require additional testing in a prospective trial reporting outcomes following the clinical use of sex-specific thresholds [97].

Interesting data come from a prospective cohort of 1126 patients with suspected ACS. Classification according to sex-specific threshold levels for hs-cTnI (34 ng/L in men, 16 ng/L in women versus 26 ng/L as standard reference value) led to an increase in the number of MI diagnosed in women (from 16% to 22%) whereas the effect on men was less relevant. Furthermore, female patients with levels of hs-cTnI of 17-26 ng/L presented sixfold rates of death or recurrent MI at 1 year when compared to women with hs-cTnI ≤16 ng/L (23% versus 4%). Similar rates of 1-year outcomes were observed when comparing women in the 17-26 ng/L group with women with hs-cTnI above the standard reference value, suggesting that a sex-specific approach improved the identification of high-risk females in this cohort [98]. While there is further evidence in support of the higher reclassification rate observed in women when using this approach [99], a subanalysis of the GUSTO-IV trial failed to identify an improved risk prediction. Notably, in this study females accounted for less than 40% of the main cohort [100]. Likewise, in a study which pooled cohorts from two randomized controlled trials, small reclassification rates occurred when using sex-specific cut-offs, thus leading to no-impact on the prognostic performance of hs-cTnI. However, the small ratio of females enrolled (31%) and the population selection criteria (patients presenting with typical ischemic symptoms) represent important biases to keep into account when translating these findings to the real world [101].

## 6. Conclusions

The influence of patients' characteristics on biomarkers and their application to clinical decisions are gaining increasing importance and consideration in modern medicine. Sex, among others, represents one of the most important factors to consider when dealing with markers such as hs-cTn, whose concentrations can overturn clinical approaches and workups.

Our review highlights some key aspects. Firstly, algorithms proposed for the work-up of ACS in the ED do not consider personal characteristics, thus potentially leading to underdiagnosis and inequality of care. Concerns were raised regarding the possible impact of sex on this issue, yet no definitive evidence is available. Secondly, current evidence clearly shows a significant difference in hs-cTn concentrations and reference limits between men and women. Among healthy people 99^th^ percentile values were found to be consistently lower in females, even if point values broadly fluctuate across studies and seem to be closely related to their reference population. Thirdly, data on the real-world performance of these sex-specific cut-offs is far more unclear. While some evidence points to potential benefits in the classification of high-risk women, several studies failed to demonstrate an advantage in terms of prognosis and clinical management [91–93, 102], thus not supporting their implementation in clinical practice. Some remarks, however, are mandatory: most of these studies investigated a single set of sex-related cut-offs, making it difficult to establish which set (if any) has the better performance in terms of risk-prediction and prognosis. Moreover, rates of reclassification (i.e.: patients with a diagnosis upgraded from UA to NSTEMI) are generally low, partly due to the narrow gap between the standard cut-off and the threshold applied to women, thus leading to a scarce impact on the overall prognosis. This is also confirmed by a recent meta-analysis, which reported the mean between-sex differences for hs-cTn in several large populations, as well as showing that the gap between standard and sex-specific thresholds is narrower for hs-cTnT, for which the mean difference of sex-specific cut-offs is close to the limit of detection [103]. In our opinion, according to the data observed and the slight differences observed between sexes in terms of hs-cTn upper reference limits [103], definitive conclusions could only be drawn on the basis of larger studies involving a higher number of patients and a more representative proportion of females, who now account for roughly 35-40% in most studies. Furthermore, in the context of MI, it is conceivable that most patients will present high levels of hs-cTn. The application of sex-tailored cut-offs then, despite the slight reclassification rate, could still improve the management of a sizeable cluster of patients. Fourthly, mechanisms underlying this discrepancy have not yet been fully explained: although some hypotheses have been reported and several factors outlined, a more thorough comprehension is required to understand if sex-related cut-offs could really impact the management of ACS in the ED, and why. For example, women exhibit higher rates of type-2 MI [104, 105] and microvascular CAD [106], and the extent to which these differences could impact hs-cTn diagnostic performance (e.g.: affecting its release kinetics or its peak values) is still a matter of concern. Further investigations are required to explore and shed some light on these open issues.

In conclusion, current literature strongly identifies the existence of sex-driven differences in hs-cTn levels in reference populations. The adoption of sex-specific cut-offs is still debated and knowledge on the potential positive effect than this could have on the prognosis of ACS in women is partial. Caution is mandatory due to lacking data on pathophysiology and further studies are required to clarify whether and why the adoption of sex-oriented cut-offs could lead to better management of ACS in women.

## Figures and Tables

**Table 1:** Studies reporting 99^th^ percentile values for hs-cTnT in different reference populations. †=median [IQR];  ^a^=only range provided; CI: confidence interval; cTn: cardiac troponin; eGFR: estimated glomerular filtration rate; UK: United Kingdom; US: United States; ARIC: Atherosclerosis Risk in Communities study; CHS: Cardiovascular Health Study; DHS: Dallas Heart Study.

Study	Study objective	Year	Location	Study population	Age, mean ± SD	Population, according to sex		99^th^ percentile (ng/L) [95% CI]		Comments
						Males (%)	Females (%)	Males	Females	
**hs-cTnT (Roche Diagnostics)**										

Collinson et al. [40]	To determine the effect of patient selection on the 99^th^ reference percentile	2012	UK	545	58 [51-67]^†^	259 (47.5)	286 (52.5)	22.8	12.8	Reference population selection based on: medical history, biomarkers and cardiac imaging

Apple et al. [61]	To systematically assess 99^th^ percentiles of cTn concentrations in a single population for a large number of assays	2012	US	524	18-64^a^	272 (52)	252 (48)	20	13	Reference population selection based only on health questionnaire interviews

Giannitsis et al. [62]	To validate the hs-cTnT assay	2010	US	616	44 ± 13.8	309 (50.2)	307 (49.8)	14.5	10	Reference population selection based only on medical records

Saenger et al. [75]	To evaluate the analytical performance of the hs-cTnT assay in a multicenter, international trial	2011	US, Europe	533	37	268 (50.3)	265 (49.7)	15.5	9	Reference population selection based only on medical records

				1600 (whole cohort)	61 ± 14	872 (54.6)	728 (45.4)	21.8 [19.8-33.9]	16.3 [12.4-18]	Reference population selection based on medical history, biomarkers and cardiac imaging; population stratified by age; Various subpopulations included: high heterogeneity and reference population unable to achieve the recommended statistical power to determine the 99^th^ percentile for each subgroup
Franzini et al. [76]	To determine the 99^th^ upper reference limit for cTnT in Italian apparently healthy subjects	2015	Italy	553	<20	270 (48.8)	283 (51.2)	10.9 [6.7-20.4]	6.8 [5.2-8.9]	
				872	20-64	503 (57.7)	369 (42.3)	23.2 [17.3-34.1]	10.2 [8.5-21.9]	
				175	>65	99 (56.6)	76 (43.4)	36.8 [21.7-37]	28.6 [17.6-28.6]	

Mingels et al. [77]	To study the improvements made by new hs-cTn assays in detecting exercise-induced cTn release	2009	US	479	51 [26-71]^†^	264 (55.1)	215 (44.9)	16	8	Reference population selection based only on medical records

				1540 (whole cohort)	57 ± 8	733 (47.6)	807 (52.4)	16 [15-17]	12 [10-14]	Reference population selection based on: medical history and biomarkers; population stratified by age
Kimenai et al. [78]	To assess sex-specific and age-specific 99^th^ percentile upper reference limits of hs-cTnT and hs-cTnI in a single reference cohort	2016	Netherlands	283	40-49	120 (42.4)	163 (57.6)	16 [10-17]	12 [7-16]	
				946	50-64	443 (46.8)	503 (53.2)	14 [13-16]	12 [9-15]	
				311	65-75	170 (54.7)	141 (45.3)	28 [19-40]	27 [12-36]	

Koerbin et al. [79]	To evaluate the analytical characteristics of the hs-cTnT assay	2010	Australia	111	25-74^a^	62 (55.9)	49 (44.1)	12.9	11	Reference population selection based on medical history, biomarkers and cardiac imaging

				DHS: 1978	43.2 ± 9.6	873 (44.1)	1105 (55.9)	17 [13-50]	11 [7-15]	Reference population selection based on: progressive cohorts restriction based on clinical history, imaging and/or laboratory tests
Gore et al. [44]	To determine the 99^th^ percentile values in three large community-based subcohorts, restricted by healtiness criteria	2014	US	ARIC: 7575	61 ± 9	2972 (39.2)	4603 (60.8)	26 [23-30]	15 [14-17]	
				CHS: 1374	72 ± 6	489 (35.6)	885 (64.4)	34 [26-42]	24 [18-35]	

Mueller et al. [80]	To assess 99^th^ percentile in a blood donors population	2016	Austria	402	35 [25.9-45.1]	259 (64.4)	143 (35.6)	13.9	11.3	Reference population selection based on: no overt cardiovascular disease, eGFR>90 ml/min

Ungerer et al. [81]	Determine and compare 99^th^ percentile cut-offs of 3 cTn assays in a cohort of blood donors	2016	Australia	2004	Male: 43.7 [30.7-54.3]	1299 (64.8)	705 (35.2)	31.3 [90% CI: 25.0-57.5]	20.2 [90% CI: 9.9-51.7]	Reference population selection based on: health questionnaire
					Female: 33.2 [24.6-50.32]					

Yang et al. [95]	Establish 99^th^ percentile in a healthy Chinese population	2016	China	1725	Male: 54 ± 20	818 (47.4)	907 (52.6)	Several according to age	Several according to age	Reference population selection based on clinical history, physical examination, lab tests
					Female: 54 ± 19					

Monneret et al. [107]	Establish age and sex specific 99^th^ percentile in patients without CKD	2018	France	2707	Male: 62 [52-70]	1548 (57.2)	1159 (42.8)	Several according to age	Several according to age	Reference population selection based on age partitioning and outliers removal. Cut-off obtained with an analytical imprecision-based approach
					Female: 63 [48-75]					

Welsh et al. [108]	Evaluating the influence of several variables, including sex, on the 99^th^ percentile levels of hs-cTnT and hs-cTnI	2018	Scotland	19501	35-65^a^	8126 (41.7)	11375 (58.3)	Several according to age	Several according to age	Reference population selection based on general population; health questionnaire; lab tests

**Table 2:** Studies reporting 99^th^ percentile values for hs-cTnI in different reference populations. †=median [IQR];  ^a^=only range provided; BMI: body mass index; BNP: brain natriuretic peptide; CI: confidence interval; cTn: cardiac troponin; eGFR: estimated glomerular filtration rate; HbA1c: glycated hemoglobin; US: United States.

Study	Study objective	Year	Location	Study population	Age, mean ± SD	Population, according to sex		99^th^ percentile (ng/L) [95% CI]		Comments
						Males (%)	Females (%)	Males	Females	
**hs-cTnI (Abbott Diagnostics)**										

Apple et al. [61]	To systematically assess 99^th^ percentiles of cTn concentrations in a single population for a large number of assays	2012	US	524	18-64^a^	272 (52)	252 (48)	36	15	Reference population selection based only on health questionnaire interviews

Koerbin et al. [63]	To assess analytical characteristics and to apply the assay to a population of apparently cardiovascular disease-free people	2012	Australia	497	20-84^a^	231 (46.5)	266 (53.5)	14	11	Reference population selection based on medical history and biomarkers

Aw et al. [85]	To determine 99^th^ percentile reference values in a large Asian cohort	2013	Asia	1120	50.4 ± 8.2	597 (53.3)	523 (46.7)	32.7	17.9	Reference population selection based on: medical history

Krintus et al. [27]	To assess 99^th^ percentile for hs-cTnI in a large multicenter European cohort	2015	Europe	1769	49 [18-60]^†^	776 (43.9)	993 (56.1)	27	11.4	Reference population selection based on blood donors, health questionnaires and no overt cardiovascular disease

Omland et al. [86]	To assess sex-related differences in hs-cTnI distribution across sexes	2015	Norway	8099	Males: 50.2 ± 17.1	3670 (45.3)	4429 (54.7)	34.8 [26.3-49.4]	18.7 [14.8-23.1]	Reference intervals are reported for women and men without history of major cardiovascular disease or risk factor
					Females: 49.7 ± 16.4					

Zeller et al. [87]	To assess sex-specific 99^th^ percentile reference values in a large German-based cohort	2015	Germany	4138	50 [42 − 61]^†^	2098 (50.7)	2040 (49.3)	33.1 [28.3-45.8]	19.9 [16.1-23.9]	Reference population selection based on different criteria with several subgroups reported (here the overall)

				1535 (whole cohort)	57 ± 8	733 (47.6)	807 (52.4)	20 [14-22]	11 [8-13]	Reference population selection based on: medical history and biomarkers; population stratified by age
Kimenai et al. [78]	To assess sex-specific and age-specific 99^th^ percentile upper reference limits of hs-cTnT and hs-cTnI in a single reference cohort	2016	Netherlands	283	40-49	120 (42.4)	163 (57.6)	13 [5-15]	12 [10-14]	
				944	50-64	441 (46.7)	503 (53.3)	22 [13-23]	9 [6-14]	
				308	65-75	168 (54.5)	140 (45.6)	20 [13-25]	13 [10-13]	

Mueller et al. [80]	To assess 99^th^ percentile in a blood donors population	2016	Austria	402	35 [25.9-45.1]^†^	259 (64.4)	143 (35.6)	39.0	23.5	Reference population selection based on: no overt cardiovascular disease, eGFR > 90 ml/min

Ji et al. [88]	To assess 99^th^ percentile values in a Korean cohort	2016	South Korea	854	49.8 ± 10.2	426 (49.9)	428 (50.1)	20	19	Reference population selection based on clinical history and laboratory tests (eGFR, HbA1c, BNP)

Li et al. [89]	To assess 99^th^ percentile for hs-cTnI in a Chinese-based population	2017	China	1485	36 ± 13	731 (49.2)	754 (50.8)	31.1	22.7	Reference population selection based on: clinical history, BMI, renal function

Welsh et al. [108]	Evaluating the influence of several variables, including sex, on the 99^th^ percentile levels of hs-cTnT and hs-cTnI	2018	Scotland	19501	35-65^a^	8126 (41.7)	11375 (58.3)	Several according to age	Several according to age	Reference population selection based on general population; health questionnaire; lab tests

**hs-cTnI (Beckman Coulter)**										

Apple et al. [61]	To systematically assess 99^th^ percentiles of cTn concentrations in a single population for a large number of assays	2012	US	524	18-64^a^	272 (52)	252 (48)	52	23	Reference population selection based only on health questionnaire interviews

**hs-cTnI (Singulex)**										

Apple et al. [41]	To determine 99^th^ percentile reference value for hs-cTnI assay	2010	US	348	18-76^a^	147 (42.2)	201 (57.8)	16.6	9.4	Reference population selection based only on health questionnaire interviews

Bossard et al. [90]	To assess factors related to hs-cTnI levels in a healthy young population without overt cardiovascular diseases	2016	Liechtenstein	2077	36.7 [31.1-40.2]^†^	975 (46.9)	1102 (53.1)	15.8	5.1	Reference population selection based on: clinical records and absence of comorbidities

**hs-cTnI (Siemens)**										

Apple et al. [61]	To systematically assess 99^th^ percentiles of cTn troponin concentrations in a single population for a large number of assays	2012	US	524	18-64^a^	272 (52)	252 (48)	81	42	Reference population selection based only on health questionnaire interviews

McKie et al. [42]	To define hs-cTnI reference values and determinants in the general community, in a healthy reference cohort, and in subsets with diseases	2013	US	565	54 [50-61]^†^	260 (45)	305 (54)	55 [32-124]	33 [22-155]	Reference population selection based on medical history, biomarkers and cardiac imaging

**Table 3:** Studies reporting performance and prognostic impact of sex-specific cut-offs in different populations. MACE: major adverse cardiovascular events; MI: myocardial infarction.

Study	Year	Patients	Women (%)	Cut-off applied (ng/L)		Comments
				Men	Women	
**hs-cTnT (Roche Diagnostics)**						

Mueller-Hennessen et al. [92]	2016	1282	477 (37%)	15.5	9.0	Sex-specific cut-offs increased MI diagnosis in women (from 17% to 23%) but this did not affect outcomes

				15.5	9.0	Reclassification occurred in only 3 patients; no effects on outcomes. Tested three different sets of sex-specific cut-offs
Rubini Gimenez et al. [91]	2016	2734	876 (32%)	17.0	9.0	
				12.0	16.0	

				16.0	9.0	Using sex-specific cut-offs, the prevalence of MI would increase by 3.3% in women. Sex-specific cut-offs did not improve risk prediction, but the study identified an increase of risk in women starting at 10-12 ng/L instead of 14 ng/L.
Eggers et al. [93]	2016	57556	22027 (38%)	26.0	15.0	
				34.0	24.0	

Mueller et al. [99]	2018	3588	1643 (46%)	16	9	Sex-specific cut-offs increased myocardial injury diagnosis in 11% of women compared to a 4% decrease in men

McRae et al. [94]	2018	7130	3199 (45%)	Several combinations according to sex		Implementation of sex-specific cut-offs improved specificity of hs-cTnT in the diagnostic approach of ACS

Yang et al. [95]	2016	812	376 (46%)	Several according to age and sex		Sex-specific cut-offs were calculated in a healthy Chinese cohort and further stratified for age

**hs-cTnI (Abbott Diagnostics)**						

Shah et al. [96]	2018	48282	22562 (47%)	34	16	Sex-specific cut-offs for an hs-cTnI assay, compared to a contemporary cTnI assay, led to a two-fold myocardial injury reclassification rate in women; no difference in 1-year outcomes among reclassified patients treated according to cTnI vs hs-cTnI levels

Shah et al. [98]	2015	1126	504 (45%)	34	16	Sex-specific cut-offs increase MI diagnosis in women (from 16 to 22%) while having small effects on men

Mueller et al. [99]	2018	3588	1643 (46%)	34	16	Sex-specific cut-offs increased myocardial injury diagnosis in 6% of women compared to a 3% decrease in men

Cullen et al. [97]	2016	2841	1180 (41%)	34	16	Small amount of women and men reclassified using sex-specific thresholds, thus improving identification of women at long-term (1 year) risk for MACE

Eggers et al. [100]	2014	2750	1073 (39%)	24.8	16.6	Sex-specific cut-offs were derived from a reference population recruited for the purposes of the study. Sex-specific cut-offs did not show improvement in the identification of more at-risk patients; however higher concentrations of troponins show stronger predictive value in women than men

Bohula May et al. [101]	2014	4695	1460 (31%)	34	16	Population presenting with typical ischemic symptoms. Using sex-specific thresholds, only 6 patients were reclassified; no improvement in prognostic performance.

## Abbreviations

ACS: Acute coronary syndrome
CAD: Coronary artery disease
CK: Creatine Kinase
cTn: Cardiac Troponin
ECG: Electrocardiography
ED: Emergency Department
ESC: European Society of Cardiology
hs-cTnI: High sensitivity cardiac troponin I
hs-cTnT: High sensitivity cardiac troponin T
MACE: Major adverse cardiac events
MI: Myocardial infarction
NSTE-ACS: Non-ST-elevation acute coronary syndrome
NSTEMI: Non-ST-elevation myocardial infarction
UA: Unstable angina.
